# A processive endoglucanase with multi-substrate specificity is characterized from porcine gut microbiota

**DOI:** 10.1038/s41598-019-50050-1

**Published:** 2019-09-20

**Authors:** Weijun Wang, Tania Archbold, Joseph S. Lam, Matthew S. Kimber, Ming Z. Fan

**Affiliations:** 1 Department of Animal Biosciences, University of Guelph, Guelph, Ontario N1G 2W1 Canada; 2 Department of Cellular and Molecular Biology, University of Guelph, Guelph, Ontario N1G 2W1 Canada

**Keywords:** Environmental biotechnology, Applied microbiology

## Abstract

Cellulases play important roles in the dietary fibre digestion in pigs, and have multiple industrial applications. The porcine intestinal microbiota display a unique feature in rapid cellulose digestion. Herein, we have expressed a cellulase gene, *p4818Cel5_2A*, which singly encoded a catalytic domain belonging to glycoside hydrolase family 5 subfamily 2, and was previously identified from a metagenomic expression library constructed from porcine gut microbiome after feeding grower pigs with a cellulose-supplemented diet. The activity of purified p4818Cel5_2A was maximal at pH 6.0 and 50 °C and displayed resistance to trypsin digestion. This enzyme exhibited activities towards a wide variety of plant polysaccharides, including cellulosic substrates of avicel and solka-Floc^®^, and the hemicelluloses of β-(1 → 4)/(1 → 3)-glucans, xyloglucan, glucomannan and galactomannan. Viscosity, reducing sugar distribution and hydrolysis product analyses further revealed that this enzyme was a processive endo-β-(1 → 4)-glucanase capable of hydrolyzing cellulose into cellobiose and cellotriose as the primary end products. These catalytic features of p4818Cel5_2A were further explored in the context of a three-dimensional homology model. Altogether, results of this study report a microbial processive endoglucanase identified from the porcine gut microbiome, and it may be tailored as an efficient biocatalyst candidate for potential industrial applications.

## Introduction

Plant lignocellulosic biomass, mainly including cellulose, hemicelluloses, lignin and pectin, is the largest renewable natural resource for the potential production of biofuel, biomaterials, and chemical feedstocks^1^. This biomass material also constitutes the major portion of dietary fibers that are utilized by various animal species^2^. The anaerobic fermentation of dietary fibers by gut microbiota is critical in maintaining the health of host animals. This fermentation assists in the supply of energy, vitamins and minerals, detoxification of noxious compounds, modulation of the host’s immune system, and protection of the host from colonization by pathogenic microorganisms^2–5^. On the other hand, some specific types of dietary fibers, such as cellulose and lignin, have been considered as an anti-nutritional factor particularly for monogastric food production animals^6^. Cellulose is the most abundant plant polysaccharide and the major component of dietary fibers. Cellulases are a group of enzymes that decompose cellulosic material, typically including endoglucanases, exoglucanases and β-glucosidase, and play concerted roles in cellulose digestion in animals^2^. Moreover, cellulases are also widely used in multiple industries^7^, including the food, textile, laundry detergent, pulp and paper, as well as in the livestock production sectors for improving feed conversion efficiency and enteric health^8^. For this reason, tremendous efforts have been directed to the research on cellulolytic enzymes.

Metagenomic mining is a powerful tool in the search for novel cellulose-degrading enzymes, either through enzyme functional screening^9^ or through gene cataloguing by high-throughput sequencing^10,11^. Functional screening of expression libraries has the advantage over gene cataloguing approaches in that enzyme activity is assayed directly, and thus allows the discovery of new enzyme functionalities and novel enzyme families with no sequence similarity to previously characterized enzymes^9^. To date, function-based metagenomic screening has unearthed a diversity of cellulases from the gut microbiota of various species^12–14^ and environmental samples^15,16^.

Gut microbiomes encompass a diverse repertoire of cellulase genes^17^. Metagenomic analyses have revealed tremendous sequence diversity for cellulases from the gut microbiomes of humans, rodents, and ruminants^2,18,19^. In contrast, metagenomic analyses and functional characterization of gut microbial cellulases in monogastric food animals are relatively limited. Pigs are known for their ability to readily adapt to a wide variety of high-fiber diets^20^. Digesta passage rate throughout the porcine gut system is relatively rapid^20^, as further compiled in Table S1. Thus, a unique gut microbiota has been established in the pig for dietary fibre degradation^21,22^. Our previous functional metagenomic screen of the pig cecum and colon microbiomes identified 18 carbohydrate-active enzyme (CAZyme) genes in total, eleven of them were co-localized and formed four function-related gene clusters coding enzymes potentially for cellulose, β-(1 → 4)-D-mannan and pectin degradation^23^. A recent metagenomic analysis of porcine microbiome also detected 29 families of CAZyme sequences likely associated with cellulose, xylan and pectin degradation^24^.

Relative to the vast amount of sequence data generated through metagenomic analyses, biochemical characterization data for these candidate cellulolytic enzymes is scarce. Given the limitation of computational prediction in the detail protein function from sequences, mapping biochemical function to an exponentially increasing number of deposited enzyme sequences represents one of the major challenges in the post-metagenomic analysis era^25^. Indeed, most of the current biochemical information regarding cellulolytic enzymes was obtained from the isolated and pure cultured microorganisms^26,27^. Considering the challenge in culturing most of animal gut microorganisms, the biochemical characterization for the cellulolytic enzymes identified directly from gut microbiome will add novel insights into the molecular mechanism of dietary fiber digestion in animal hindgut. We thus set out to mine novel cellulases from the pig gut microbiome by a metagenomic functional screening. One of the discovered cellulase genes (*p4818Cel5_2A*) was further expressed for the detail biochemical characterization. The resulting enzyme proved trypsin resistant, and have a broad substrate specificity towards a variety of plant polysaccharides. More specifically, it acted as a monomodular and processive β-(1 → 4)-endoglucanase in hydrolyzing cellulose into cellobiose and cellotriose as the primary end products.

## Results and Discussion

### Diet induction and functional metagenomic screening identified a novel cellulase gene from the pig gut microbiome

The porcine hindgut microbiota is a unique ecosystem, capable of rapid cellulose degradation (Table S1)^21,22^. A metagenomic plasmid expression library was previously constructed from the pooled hindgut microbiota of six grower pigs, which were enriched for crystalline cellulose degradation by feeding with a diet containing 10% Solka-Floc^®^ as the sole fiber source (Table S2) for 28 days, as detailed in our previous study^23^. The functional screening against this library (Fig. S1A,B) had resulted in the identification of 18 CAZyme genes, and eleven of them were co-localized and formed four function-related gene clusters^23^ (Fig. S2).

**Figure 1:**
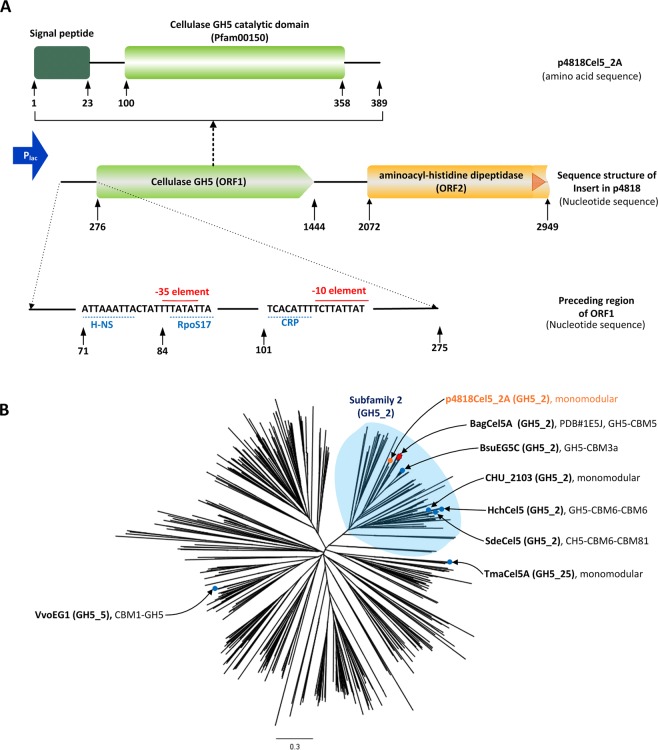
Organization of the insert from the p4818 positive clone and phylogenetic analysis of p4818Cel5_2A with other characterized members of the GH5 family. (**A**) Organization of the insert from positive clone of p4818. Two open reading frames (ORFs) were identified within this insert. The predicted −10 and −35 promoter elements are shown in red above the sequences and the identified transcription factor binding sites are underlined in blue. The prediction for bacterial promoters was performed using the online tool of BPROM-Prediction^67^ against 274 bp sequence proceeding to the cellulase p4818Cel5_2A coding region. Enzyme modules were identified using the Simple Modular Architecture Research Tool (SMART, http://smart.embl-heidelberg.de/). (**B**) Phylogenetic analysis of p4818Cel5_2A with sequences of characterized GH5 enzymes. All sequences were aligned using MUSCLE in Geneious version 8.0.5. The tree was constructed using Geneious Tree Builder version 8.05. The other reported processive GH5 endoglucanases were indicated by blue dot, and the sequence of template structure (PDB#1E5J) was indicated by red prism.

One previously identified positive clone^23^, referred to as p4818 (Fig. S2), was sequenced by primer walking. The DNA insert is 2949-bp long with 39% G + C content (GenBank accession number, MH373350), containing two tandem open reading frames (ORFs). The ORF at 3′ side was predicted as a partially C-terminal truncated aminoacyl-histidine dipeptidase with a closest homologue of an aminoacyl-histidine dipeptidase from *Faecalibacterium prausnitzii* KLE1255 (Genbank accession number: ZP07799867, sequence identity of 60%). The other ORF at 5′ side was predicted to encode a cellulase with the closest homologue being an uncharacterized endoglucanase from *Eubacterium rectale* DSM 17629 (Genbank accession number: CBK89462, sequence identity of 51%) (Fig. 1A). Both bacterial species belong to the class *Clostridia*. However, through the megablast with the entire insert nucleotide sequence against nucleotide (nr/nt) databases, no sequence hit could be found for having the significant nucleotide sequence similarity to the inquiry, suggesting that this insert sequence was likely derived from an unsequenced *Clostridia* species. Furthermore, we performed an amino acid sequence search for this cellulase against EBI-metagenome database - Mgnify (https://www.ebi.ac.uk/metagenomics; search parameters: E < 0.01, gap penalty of 0.02 for open and 0.04 for extend). This resulted in 2416 hits, with the closest homologue being MGYP000014238623 (amino acid sequence identity of 60.3%) from the human digestive sub-datasets in “Host-associated Biome Database (v.2018_06)”. The similar search against animal sub-dataset (v.2018_06) resulted in 135 hits, with a closest homologue being MGYP000572419700 (amino acid sequence identity of 57.0%). Together, this indicates that homologues of p4818Cel5_2A (a member of GH5_2, see the result below) are widely present in the gut microbiomes of human and other animal species. Consistently, GH5 family enzyme sequences were found to be abundant among human microbiomes^19^. Immediately upstream of the start codon of this putative cellulase, the potential −35 (TTATA) and −10 (TCTTATTAT) promoter elements were identified. Canonical binding sites for three transcription factors were also found in this region, including CRP (cAMP receptor protein, also known as catabolite activator protein; CAP), H-NS (histone-like nucleoid structuring protein) and RpoS (RNA polymerase, δ^S^ subunit) (Fig. 1A). CRP is a cAMP-activated global transcription factor for catabolite repression particular in microbial carbon and energy metabolism^28^. While, RpoS is a central regulator of the general stress response, and its association with CRP has been found in microbial metabolism regulation^29^. Lastly, H-NS is a component of bacterial chromatin, and influences gene expression at both local and global scale^30^. Together, these observations imply a coordinated regulation in the expression of this novel cellulase gene under pig hindgut environment.

This predicted cellulase ORF comprises 1170 nucleotides, encoding a protein of 389 amino acids (Fig. 1). The presence of an N-terminal signal peptide (1–23 amino acids) is consistent with the expected extracellular location of this enzyme. The cellulase was predicted to be a member of the family 5 glycosyl hydrolases (GH5) (CAZy database, http://www.cazy.org/), based on its clustering with known GH5_2 subfamily sequences in a phylogenetic analysis^31^ (Fig. 1B), and thus belongs to subfamily 2 in GH5 family. Accordingly, it was designated as p4818Cel5_2A cellulase. Although the catalytic domain(s) of cellulases are often associated with specific carbohydrate-binding modules (CBMs)^32^, no canonical CBM is recognizable in p4818Cel5_2A sequence, and thus this enzyme is a monomodular cellulase.

### Expression, purification and characterization of p4818Cel5_2A cellulase

**Figure 2:**
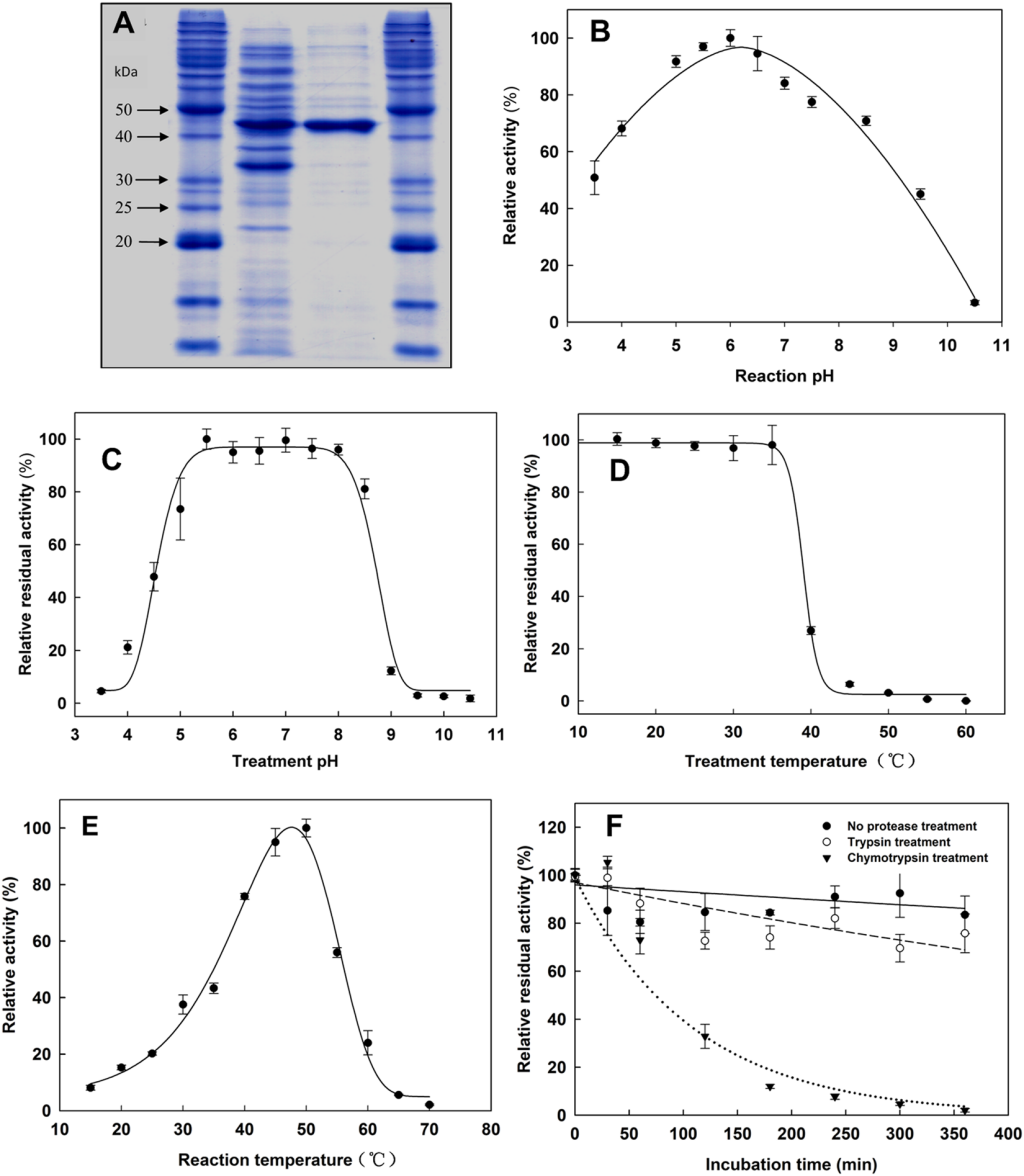
Purification and general properties of p4818Cel5_2A enzyme. (**A**) Purification of p4818Cel5_2A, Lanes 1,4: Molecular weight standards; lane 2: Crude extract; and lane 3: Ni-NTA purified p4818Cel5_2A cellulase. (**B**) The effect of reaction pH on p4818Cel5_2A activity on CMC. The activity was presented as relative activity(%) to the specific activity of 1816.0 µmol/(mg protein · min) measured at optimum pH 6.0. A constant ionic strength buffers containing 100 mM Tris, 50 mM acetic acid and 50 mM MES over a pH range from 4.0 to 10.0 were used. (**C**) pH stability of the purified p4818Cel5_2A enzyme activity relative to % of the residual activity after the incubation under pH 7.0. (**D**) The relative residual activity of purified p4818Cel5_2A after treatment at the indicated temperature relative to % of the residual activity after the incubation at 15 °C. (**E**) The effect of reaction temperature on p4818Cel5_2A activity relative to % of the activity measured at 50 °C. (**F**) Resistance of p4818Cel5_2A activity to porcine trypsin and bovine chymotrypsin treatment relative to % of the p4818Cel5_2A activity measured without any protease treatments. The p4818Cel5_2A cellulase in the final concentration of 50 µg/ml was incubated with either 5000 U/ml trypsin (BAEE unit) or 200 U/ml chymotrypsin (BTEE unit) at 37 °C in 100 mM MES buffer (pH 6.0). The purified P4818Cel5_2A cellulase of 1.6 µg and 1.0% carboxymethyl cellulose (CMC) were used in all assays for (**B–E**). Values were expressed as means ± SE, n = 3.

GH5 enzymes display a variety of hydrolytic activities^31^, making sequence-based function predictions challenging for this family. The sequence encoding the mature form of p4818Cel5_2A (with the 1–23 amino acids signal peptide sequence removed) was inserted into a pET28a expression vector, fused in frame with an N-terminal His-tag. The recombinant p4818Cel5_2A was purified with a typical yield of 19 mg protein for per liter of *E. coli* culture. SDS-PAGE analysis of the purified protein depicted a protein with an apparent molecular mass of 43 kDa (Fig. 2A), which is consistent with its theoretical molecular mass of 43.4 kDa.

The p4818Cel5_2A activity on carboxymethyl cellulose (CMC) exhibited a reaction pH optimum at 6.0, retaining >70% of its maximal activity from pH 4.0 to 8.5 (Fig. 2B). It retained over 90% of its original activity after storage for 24 h at 4 °C in the pH range from 5.5 to 8.0 (Fig. 2C). The enzyme retained ~95% and ~30% activity after 30 min incubation under 35 °C and 40 °C, respectively; however, 95% activity was lost at temperatures >45 °C (Fig. 2D). Interestingly, the optimal reaction temperature of p4818Cel5_2A for 10-min reaction was at 50 °C (Fig. 2E), a temperature where the isolated enzyme was unstable, possibly indicating that the binding of the enzyme to CMC significantly increased the enzyme’s thermostability. In addition, the effects of selected divalent metal ions and chemicals on the enzyme activity were also examined (Table S3). The p4818Cel5_2A activity towards CMC was inhibited (*P* < 0.05) in the presence of 1.0 mM Ni^2+^ (by 12%), Cu^2+^ (by 12%), Mn^2+^ (by 30%), Cd^2+^ (by 34%), and Zn^2+^ (by 58%), respectively. The inhibitory effects of these metal ions are likely due to their ability to bind to the active site of p4818Cel5_2A. In addition, the reducing agent of 1,4-dithiothreitol (DTT) at 5.0 mM demonstrated an activation effect (*P* < 0.05) by 27%. DTT was also previously reported to activate an endoglucanase from the anaerobic bacteria, *Clostridium thermocellum*^33^.

### p4818Cel5_2A is resistant to trypsin digestion

Enzymes secreted by microbiota in the lumen environment of cecum and the proximal colon will be exposed to residual exocrine pancreatic proteases. The ability of p4818Cel5_2A to withstand proteolysis by trypsin and chymotrypsin was therefore examined. p4818Cel5_2A retained >70% of its original activity on CMC after 6 h of incubation with trypsin at 5,000 U/ml under 37 °C, whereas it lost 95% of its activity after an analogous incubation with chymotrypsin at 200 U/ml (Fig. 2F). In comparison, a GH5 family cellulase from *Bacillus amyloliquefaciens* (BamCel5, Megazyme) lost ~90% of its original activity after a 5 h treatment by either trypsin or chymotrypsin under the same conditions as for p4818Cel5_2A (Fig. S3). Furthermore, the online protease cutting sites server (PeptideCutter, https://web.expasy.org/peptide_cutter/) predicts 37 and 24 cutting sites for p4818Cel5_2A by chymotrypsin and trypsin, respectively; in contrast, 28 and 27 were found for the catalytic domain of BamCel5. In general, the total number of potential cutting sites will not necessarily correlate with susceptibility to a given protease as many candidate sites are likely buried and therefore not accessible to the protease active site. Empirical identification of sites within p4818Cel5_2A that are actually cleaved by a given protease, along with structure determination will provide more insights into the molecular basis of its resistance to trypsin. Our observation is consistent with the previous report that porcine cecal digesta retained a high residual trypsin activity, while chymotrypsin activity was reduced to negligible levels^34^. To the best of our knowledge, the resistance of microbial enzymes secreted by the gut symbiotic microflora to exocrine pancreatic proteases has been scarcely reported. This feature reflects an environment-driven enzyme evolution for the resistance to proteolysis. It is of great benefit for the potential application of p4818Cel5_2A as an exogenous feed enzyme in food animal industry, as it allows more of the activity to reach its targeted site of action.

### p4818Cel5_2A has a broad substrate specificity

GH5 family of enzymes exhibited a wide variety of glycoside hydrolytic activities, including endoglucanase, endoxylanase, endomannanase, as well as exoglucanase, exomannanase, β-glucosidase, β-mannosidase and others. It is one of the largest and most diverse GH families, with more than 50 subfamilies^31^. Specifically, members in subfamily 2 of GH5 family (GH5_2) are often associated with endo-β-(1 → 4)-glucanase activity^31^. Of note, a bifunctional cellulase/chitosanase activity has been found for several members in this subfamily^35^. Herein, the substrate specificity of p4818Cel5_2A was examined against a variety of polysaccharides (Table 1). The enzyme exhibited the highest activity on β-(1 → 4)/(1 → 3)-glucan from barley, followed by CMC, hydroxyethyl cellulose (HEC), glucomannan from Konjac, xyloglucan from tamarind seed, and galactomannan from Locust bean gum. All of these polysaccharides are soluble and contain common β-(1 → 4)-glucosidic linkages in their backbone with the exception of galactomannan, which has a linear backbone of β-(1 → 4)-linked D-mannose modified (typically) sub-stoichiometrically by α-(1 → 6) linked D-galactose residues. The catalytic efficiency constant (*V*_max_/*K*_m_) further verified that barley β-(1 → 4)/(1 → 3)-glucan was the best substrate for p4818Cel5_2A among all the tested polysaccharides (Table 2; Fig. S4). In contrast, no activity was detected for curdlan [β-(1 → 3)-glucan] from *Alcaligenes faecalis* and laminarin [β-(1 → 3)-glucan backbone with β-(1 → 6)-glucosidic substitutions] from *Laminaria digitata*, indicating that p4818Cel5_2A hydrolyzes β-(1 → 4)-D-glucosidic bond. Specifically, p4818Cel5_2A activity on xyloglucan was ~170 times lower than that on β-(1 → 4)/(1 → 3)-glucan (Table 1). Xyloglucan consists of a β-(1 → 4)-linked glucan backbone that is further substituted at O6 with xylosyl residues through α-(1 → 6)-linkage, suggesting that the bulky O6-substitution at the glucose unit sterically hinders the enzyme catalysis.

**Table 1:** Specific activity of the purified p4818Cel5_2A enzyme towards various polysaccharides.

Substrate	Main linkage type and solubility	Specific activity (µmol · µmol^−1^ protein · min^−1^)
Avicel (PH101)	β-(1 → 4)-D-Glc, crystalline, insoluble	9.0 ± 0.7
Solka-Floc®	β-(1 → 4)-D-Glc, crystalline, insoluble	5.3 ± 0.4
Regenerated cellulose (RAC)	β-(1 → 4)-D-Glc, amorphous, insoluble	57.7 ± 4.0
Hydroxyethyl cellulose (HEC)	β-(1 → 4)-D-Glc, soluble	875.0 ± 75.0
Carboxymethyl cellulose (CMC)	β-(1 → 4)-D-Glc, partially substituted with carboxy methyl groups, soluble	1816.0 ± 149.0
β-Glucan from barley grain	β-(1 → 4)/(1 → 3)-D-Glc, soluble	2337.0 ± 164.0
Laminarin	β-(1 → 3)-D-Glc backbone, mainly with β-(1 → 6)-D-glucosyl substitutions, soluble	ND
Curdlan	β-(1 → 3)- D-Glc, soluble	ND
Xyloglucan	β-(1 → 4)- D-Glc backbone, mainly with β-(1 → 6)-D-Xyl substitutions, soluble	14.1 ± 0.17
Glucomannan from Konjac	β-(1 → 4)-D-Man/D-Glc, soluble	230.5 ± 21.4
Galactomannan from Locust bean gum	β-(1 → 4)- D-mannan, single D-galactosyl units attached to C-6 of some of the D-mannosyl residues, soluble	2.8 ± 0.7
Wheat arabinoxylan	β-(1 → 4)- D-Xyl backbone, partially substituted at O-2 and/or O-3 positions with L-Ara*f*	ND
Colloidal chitin	β-(1 → 4)-N-acetylglucosamine, amorphous, insoluble	ND

Enzyme activities were measured with 1.0% substrates in 100 mM MES buffer pH 6.0 under 50 °C for 10 min. The amount of the purified p4818Cel5_2A enzyme added for the activity assays on CMC, β-glucan from barley and HEC was 1.5 µg protein, 30 µg protein for xyloglucan, and 135 µg protein for all other substrates. The incubation time was 30 min for Avicel and Solk-Floc®, 60 min for larminarin and chitin, and 10 min for other substrates. Details of the activity assay were described in the Material and Methods section. All values were expressed as means ± SE, n = 3. ND: no detectable activity.

**Table 2:** Kinetics of p4818Cel5_2A towards soluble substrates.

Polymeric substrate	*K*_m(app)_ (mg/ml)	V_max_ (µmol · µmol^−1^ protein · min^−1^)	V_max_/*K*_m(app)_ (µmol · µmol^−1^ protein · min^−1^)/ (mg/ml)
Carboxymethyl cellulose (CMC)	10.9 ± 1.1	3540.0 ± 130.0	324.8
β-Glucan from barley	0.94 ± 0.15	2664.0 ± 177.0	2834.0
Glucomannan from Konjac	4.4 ± 1.5	1353.0 ± 392.0	307.5
**Synthetic substrate**	***K***_***m***_ **(mM)**	***K***_***cat***_ **(min** ^**−1**^ **)**	***K***_***cat***_ **/** ***K***_***m***_ **(min**^**−1**^ **· mM**^**−1**^**)**
*p*NP-Cellobioside	1.2 ± 0.1	97.8 ± 3.5	80.8
*p*NP-β-D-Glucopyranoside	ND	ND	ND

Activity assays were performed by the varying concentrations of corresponding substrates in 100 mM MES buffer pH 6.0 with an incubation of 10 min under 50 °C. The amount of the purified p4818Cel5_2A enzyme added for the activity assays on CMC, β-glucan from barley was 1.5 µg protein, and that for glucomannan from Konjac, *p*NP (4-Nitrophenol) - Cellobioside and *p*NP-β-D-Glucopyranoside was 135 µg protein respectively.

Kinetic parameter estimates ± SE (n = 3). ND: no detectable activity.

p4818Cel5_2A was active towards different forms of insoluble cellulosic substrates, where activity towards the regenerated amorphous cellulose (RAC) was higher than the crystalline celluloses of Avicel and Solka-Floc^®^ by 6–12 times (Table 1). These differences likely reflect the much lower substrate accessibility of Avicel and Solka-Floc^®^ to p4818Cel5_2A than that of RAC, as the crystallinity index (CI) values of Avicel (CI at 0.5–0.6) and Solka-Floc^®^ (CI at 0.4–0.7) are higher than that of RAC (CI at 0 ~ 0.04)^36^. Furthermore, the near 2-fold higher p4818Cel5_2A activity towards Avicel than Solka-Floc^®^ was likely due to the fact that Avicel has a lower degree of polymerization (DP at 150–500 glucose units) compared with Solka-Floc^®^ (DP at 750–1500 glucose units)^36^. In addition, p4818Cel5_2A exhibited a specific activity of 9.0 and 5.3 µmol glucose equivalent **· **µmol^−1^ protein **· **min^−1^ on Avicel and Solka-Floc^®^, respectively. These values are 7 to 220 folds higher than the corresponding activities of many other reported cellulases, such as a GH9 cellulase (Cel9B) and a GH51 cellulase(Cel51A) from ruminal *Fibrobacter succinogene*^37^, a GH48 cellulase(Cel48A) from *Thermobifida fusca*^38^, two GH5_2 cellulases of Cel5H and Cel5 from *Saccharophagus degradans*^39^ and from *Hahella chejuensis*^40^, respectively. Given the similar properties of Solka-Floc^®^ to pre-treated cellulosic substrates^36^, the hydrolytic activity of p4818Cel5_2A towards Avicel and Solka-Floc^®^ is thus of great interest in enhancing cellulosic biomass valorization and animal fiber digestion.

Although the enzymes from subfamily 2 in GH5 family (GH5_2) generally display endo-β-(1 → 4)-glucanase activity^31^, they can differ greatly in their substrate utilization profiles. For example, the activities towards Avicel, RAC, β-(1 → 4)/(1 → 3)-glucan and CMC were previously measured for three GH5_2 endo-β-(1 → 4)-glucanases from *Bacillus substilis* BS-5^41^, *Volvariella volvacea*^42^ and *Hahella chejuensis*^40^ respectively. Whereas, GH5_2 cellulase of CelDZ1 from hot spring microbiota was found only active on CMC and β-D-glucan, but not on Avicel^43^, and another GH5_2 cellulase from soil was found active on CMC, β-D-glucan and Avicel, while inactive to xyloglucan^44^. In comparison, p4818Cel5_2A displayed activities towards a wide variety of plant polysaccharides, including regenerated amorphous cellulose, highly crystalline cellulosic substrates (e.g., Avicel and Solka-Floc^®^), as well as the hemicelluloses of β-(1 → 4)/(1 → 3)-glucan, xyloglucan, glucomannan and galactomannan (Table 1). These tested fiber components are widely present in various animal diets and rations, such as forages, roughages, corn, barley, rice, wheat, as well as legumes (e.g. as peas, beans and soybean). Therefore, the broad substrate specificity indicates the involvement of p4818Cel5_2A in digesting a wide variety of plant polysaccharides in pig hindgut, reflecting again a diet-driven enzyme evolution in shaping substrate specificity. In addition, the broad substrate specificity of p4818Cel5_2A will be beneficial for its application as the exogenous feed enzyme in improving feed nutritional value.

### p4818Cel5_2A shows a potentially processive mode of action

**Figure 3:**
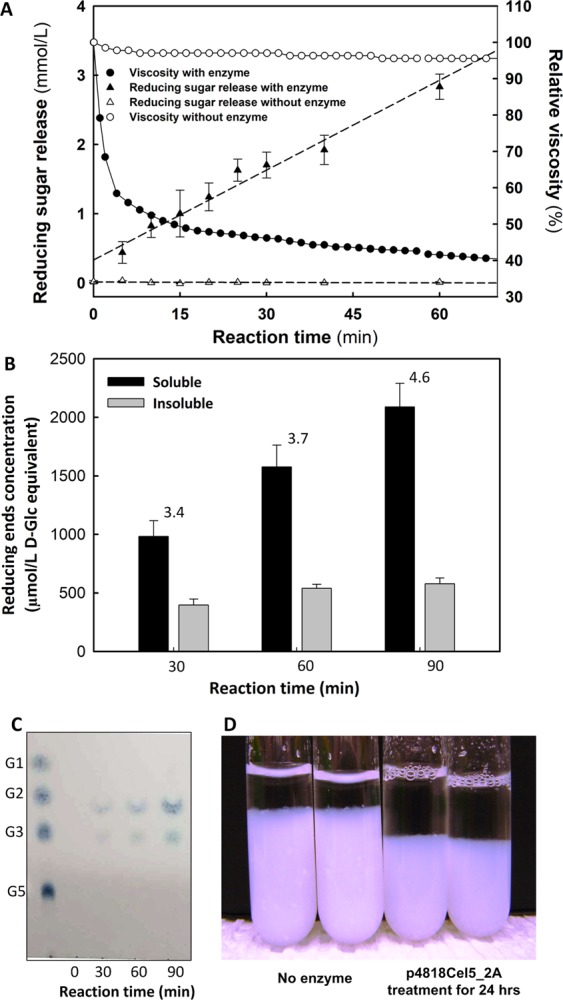
Enzyme p418Cel5_2A acts as a processive β-1,4-endoglucanase. (**A**) The response of viscosity and reducing sugar release from caboxymethyl cellulose (CMC) solution hydrolyzed by p418Cel5_2A. The purified enzyme (5.2 μg) was added to 3% CMC solution in 100 mM MES buffer (pH 6.0) to initiate the reaction in a final volume of 16 ml, and the viscosity and reducing sugar concentration (µM) in the reaction solution was monitored at 5 min intervals for 1 hour. (**B**) The processivity of p418Cel5_2A towards regenerated amorphous cellulose (RAC, 1.0%, wt/vol). The processivity is presented as the ratio of the reducing ends in soluble fraction to that in insoluble fraction, and labelled at top of the bars. (**C**) Thin layer chromatography (TLC) analysis of hydrolysis products from RAC by p418Cel5_2A. G1, D-glucose; G2, cellobiose; G3, cellotriose; and G5, cellopentose. (**D**) Solubilization of RAC (1.0%, W/V) by p418Cel5_2A for 24.0 h under 25 °C. Values were expressed as means ± SE, n = 3.

In order to understand the action mode of p4818Cel5_2A, its activity towards CMC was further examined by monitoring viscosity change and reducing sugar release over the reaction time. The viscosity of CMC assay solution declined rapidly by ~50% in the first 10 min after adding enzyme (Fig. 3A), and the entire viscosity change fitted well with a mono-exponential decay pattern (R^2^ = 0.844). In contrast, a linear increase for total reducing sugar release was observed over the reaction (R^2^ = 0.963) (Fig. 3A). Together, these results suggested that p4818Cel5_2A cellulase acts as a β-(1 → 4)-endoglucanase.

**Figure 4:**
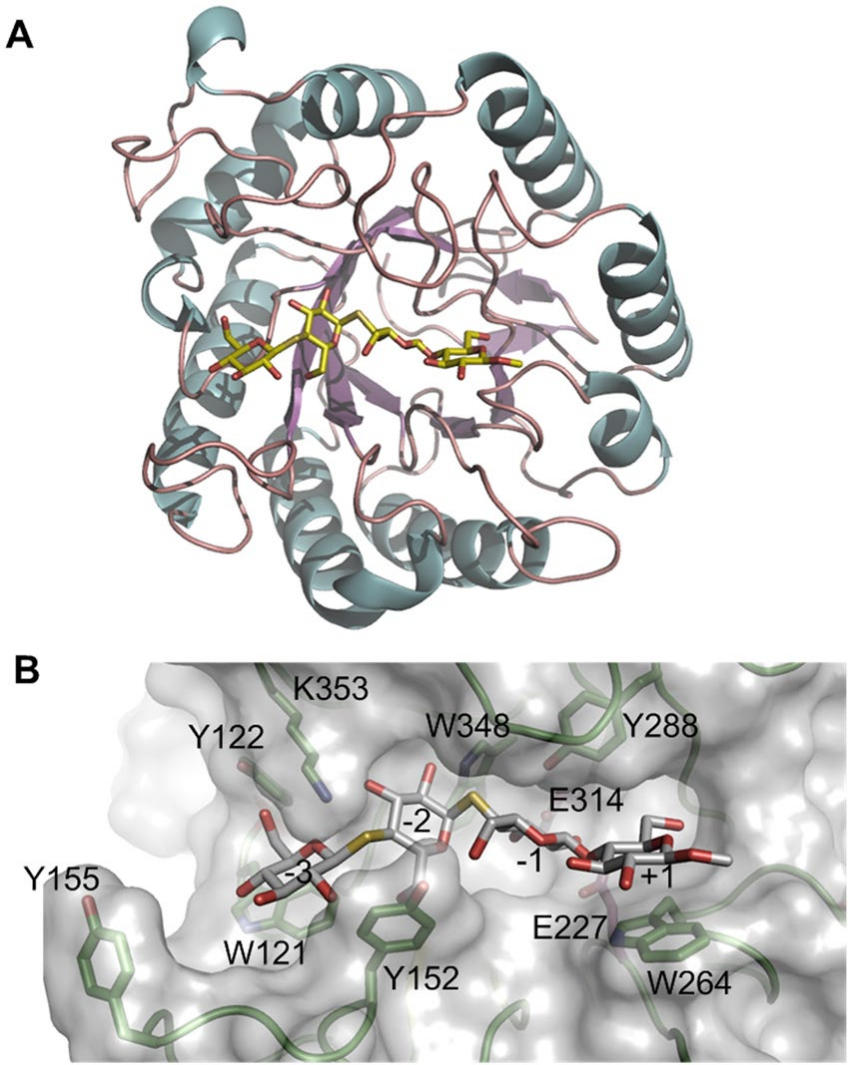
A homology model of p4818Cel5_2A. The 3-dimensional structure model for the cellulase p4818Cel5_2A was generated from residue 91 to 384 with SWISS-MODEL online server by using the crystal structure of the homologous cellulase (pdb#1E5J) as a template. (**A**) TIM barrel fold of p4818Cel5_2A with an estimated overall diameter size of 50 Å. (**B**) Predicted cellulose binding cleft and relevant aromatic amino acids involved in substrate binding. The residues of Glu227 and Glu314 were predicted as the catalytic acid/base. The cellotetraose analogue of methyl 4^II^,4^III^-dithio-α-cellobiosyl-(1 → 4)-β-cellobioside was modelled by superimposing 1E5J and is shown as sticks; select residues believed to be important in substrate binding and catalysis are shown as green sticks.

Processivity plays a critical role in cellulose enzymatic degradation, however, the assessment of cellulase processivity is challenging, as there is no straightforward approach to quantify it^45^. In this study, the processivity of p4818Cel5_2A in hydrolyzing RAC was further examined by assessing the distribution of reducing sugar ends. Specifically, the ratio of reducing ends in the soluble fraction to that in the insoluble fraction (i.e. processive ratio) was increased from 3.4 to 4.6 as the incubation time was prolonged from 30 to 90 min (Fig. 3B), generating 77.3% and 82.1% soluble reducing sugar ends, respectively. This result stands in contrast to the actions of random cutting endoglucanases, which will produce more reducing sugar ends in the insoluble faction than in the soluble fraction^46^. Further with thin layer chromatography (TLC), the predominant hydrolysis products of p4818Cel5_2A from RAC were identified as cellobiose and cellotriose with a very minor amount of glucose, irrespective of reaction time of 30, 60 or 90 min (Fig. 3C). In contrast, a wide range of cello-oligosaccharides are expected for the RAC hydrolysis by a typical random cutting endoglucanases. In total, 16.5% RAC (by dry matter) was solubilized by p4818Cel5_2A after 24 h digestion at 25 °C (Fig. 3D). Notably, cellobiose and cellotriose were also found to be the main hydrolysis products from RAC by several other processive GH5 endoglucanases^39,41^. Furthermore, p4818Cel5_2A also demonstrated activity towards *p*-nitrophenol (*p*NP)-cellobioside, a typical synthetic substrate for β-(1 → 4)-D-glucan cellobiohydrolase activity^47^, with a *K*_m_ of 1.2 ± 0.1 mM and *k*_*cat*_ of 97.8 ± 3.5 min^−1^ (Table 2). In contrast, p4818Cel5_2A did not show any detectable hydrolytic activity towards *p*NP-β-D-glucopyranoside, which is a typical synthetic substrate for measuring β-glucosidase activities and is only one glucose unit shorter than *p*NP-cellobioside. Moreover, *p*NP-β-D-glucopyranoside (10.0 mM) did not show any inhibitory effect to p4818Cel5_2A activity on *p*NP-cellobioside (1.0 mM), indicating a poor binding of *p*NP-β-D-glucopyranoside to p4818Cel5_2A enzyme (Table 2). Together, these results suggested that the subsite −2 of p4818Cel5_2A (following the nomenclature defined by Davies and colleagues^48^) played an important role in the binding *p*NP-cellobioside to the enzyme (also see Fig. 4B). In summary, our results indicate that p4818Cel5_2A is a processive β-(1 → 4)-endoglucanase for hydrolyzing cellulose into cellobiose and cellotriose as the primary end products.

In general, there are two types of processive cellulases, exocellulases and processive endoglucanases^49^. To date, most of reported processive endoglucanases are from GH families of 5, 9 and 48 whose members often exhibit multi-modular architectures, typically with a catalytic domain and one or more carbohydrate-binding modules (CBMs)^50^. Within the GH5 family, processivity has been reported for several multi-modular GH5_2 endoglucanases, including a cellulase from *Bacillus subtilis* BS-5 (BsuEG5C, GH5-CBM3)^41^, an endoglucanases from *Saccharophagus degradans* (SdeCel5H, GH5-CBM6)^39^ and an endoglucanases from *Hahella chejuensis* (HchCel5, CBM6-GH5)^40^, respectively (Fig. 1B). Of note, processive endoglucanases were also identified in the other subfamilies of the GH5 family (e.g. GH5_5 and GH5_25), including a GH5_5 endoglucanase from *Volvariella volvacea* (VvoEG1, CBM1-GH5)^42^ and a GH5_25 cellulase from *Thermotoga maritime* (TmaCel5A)^51^ (Fig. 1B). In contrast, p4818Cel5_2A is a single catalytic domain with a potential processive mode of action that hydrolyzes cellulosic substrates without any additional binding domains. To date, only a few of mono-modular cellulases have been reported to be processive, including the aforementioned TmaCel5A and a GH5_2 endoglucanase from *Cytophaga hutchinsonii* (CHU-2103)^52^ (Fig. 1B). To the best of our knowledge, p4818Cel5_2A also represents the first microbial processive endoglucanase discovered from the monogastric animal gut microbiome, which is able to hydrolyze cellulose mainly to cellobiose and cellotriose. More interestingly, the classical scheme for cellulose microbial degradation involves the synergistic action of a minimum of three enzymes: randomly cutting β-(1 → 4)-endoglucanse, β-(1 → 4)-exoglucanase and β-glucosidase. With cellobiose and cellotriose as the main hydrolysis products, the processive β-(1 → 4)-endoglucanse of p4818Cel5_2A could potentially form a novel two-enzyme pathway for degrading cellulose into fermentable monosaccharide through further coupling with β-glucosidase or β-glycoside phosphorylase in pigs’ gut.

### Structure model rationalized the monomodular catalytic features of p4818Cel5_2A

A structural model of p4818Cel5_2A was built using the crystal structure of a homologous GH5_2 cellulase (amino acid sequence identity: 47.0%) from *Salipaludibacillus agaradhaerens* (previously called *Bacillus agaradhaerens*) (BagCel5A, PDB ID#:1E5J) as template with a RMSD value of 0.354 Å^53^. This model displayed a classic triosephosphate isomerase (TIM) barrel fold with dimensions of 5.5 nm × 6.2 nm × 7.5 nm (Fig. 4A) Notably, the structure of BagCel5A showed a maximal diameter of 5.5 nm, minimum of ~4.5 nm. Erickson observed that most single modular proteins would fold into one globular domain and polypeptides larger than 50 kDa typically form two or more domains^54^. Thus, the monomodular cellulases with low molecular weight likely form various roughly spherical shapes with differing radius, while bi-modular, larger molecular weight cellulases tend to show ellipsoid or other irregular shapes^55,56^. Enzyme penetration into biomass is one of the limiting factors in biomass degradation^57^, where the pore size in substrates plays an important role. For example, the pore size in living plant cell wall was reported for a diameter ranging from 3.5 to 5.2 nm^58^, and the pretreated cellulosic substrates from lignocellulosic biomass materials have larger pore diameters, ranging between 5.1–11.0 nm^59^. The p4818Cel5_2A is modeled to have an overall globular shape (Fig. 4A) with an estimated diameter of 4.6 nm^79^. Therefore, the small size of the monomodular p4818Cel5_2A enzyme relative to multi-modular enzymes will be beneficial for its ability to penetrate into lignocellulosic biomass, and could serve as an ideal industrial biocatalyst candidate for further biological engineering.

By comparing our model with the template structure (BagCel5A) in complex with a substrate analog^53^, a narrow and extended cellulose binding cleft was identified at the C-terminal side of the p4818Cel5_2A TIM barrel (Fig. 4A). The presence of several aromatic residues, including the highly conserved W121, W348, W264 and Y288 along with Y152 and Y122, leads us to propose that this binding cleft contains at least four cellulose binding subsites (Figs 4B, S5). In particular, W264 and W121 are likely involved in hydrophobic stacking interaction with the glucose residues at subsites +1 and −3 respectively, while W348, Y152 and Y122 appear to contact glucose ring at subsite −2 or −3 through edge-on-edge interactions. Moreover, five out of the eight aforementioned amino acid residues (W121, Y122, Y152, Y158, Y290) lining substrate binding cleft are contributed by loop regions, indicating a potential flexibility of binding cleft conformation. This flexibility likely conferred p4818Cel5_2A a broad substrate profile for cleaving β-(1 → 4)-D-glucosyl bonds in a wide variety of plant polysaccharides (Tables 2, S4). In addition, the region of the binding cleft flanking the subsites from −3 to +1 is relatively open, which should allow the enzyme to accommodate xylosyl and galactosyl substitutions in the substrates xyloglucan and galactomannan, respectively. Interestingly, the side chains of Y152 and Y155 from loop region (residues 152 to 156) and K353 would further extend both sides of the substrate binding cleft in p4818Cel5_2A at binding subsites of −3 and −2 (Fig. 4B). It would likely enhance the attachment of p4818Cel5_2A to cellulose chain and allow enzyme to perform multiple rounds of catalysis before dissociating, in turn resulting in the observed processivity (Fig. 3A,B). Similarly, a well-characterized processive GH9 endoglucanase, *Tf*Cel9A, from *Themobifida fusca* has been currently shown to processively move on a polysaccharide by utilizing six sugar binding subsites, which is independent from the associated CBM^60^. Notably, residues of Y152 and Y155 in p4818Cel5_2A are not conserved across GH5 cellulases, and are often replaced by glycine, serine and leucine (Fig. S3, Table S4). A future study with structural characterization and site-specific mutagenesis will shed more light on the molecular mechanism of the catalytic features of p4818Cel5_2A enzyme.

## Conclusions

Through previous dietary crystalline cellulose induction and metagenomic screening, a novel gut bacterial cellulase gene, *p4818Cel5_2A*, identified from porcine gut microbiome, was further recombinantly expressed in the current study. This p4818Cel5_2A cellulase was resistant to trypsin digestion. Our data further showed that p4818Cel5_2A enzyme is broadly active towards a wide variety of plant polysaccharides, including cellulosic substrates of avicel and solka-Floc®, and the hemicelluloses of β-(1,4)/(1,3)-glucan, xyloglucan, glucomannan and galactomannan. Moreover, p4818Cel5_2A acted as a processive β-1,4-endoglucanase for hydrolyzing cellulose into cellobiose and cellotriose as the primary end products. It is monomodular with a modest total molecular weight, allowing it to form a compact domain. In summary, our study has revealed a novel processive β-1,4-endoglucanase with multi-substrate specificity derived from a porcine gut symbiont that enables facile cellulose digestion, but is also a promising biocatalyst candidate with the potential in multi-industrial biomass bioprocessing applications. The porcine gut microbiome has clear potential as a unique genomic resource for the discovery of biomass degradation enzymes.

## Materials and Methods

### Chemicals

QIAquick PCR purification kit and QIAamp DNA stool mini kit were all purchased from QIAGEN (Toronto, ON, Canada). AZO-CM-cellulose (AZO-CMC), curdlan, xyloglucan and GH5 family cellulase from *Bacillus amyloliquefaciens* (BamGH5A) were from Megazyme (Wicklow, Ireland). Carboxymethyl cellulose (CMC, low viscosity), hydroethyl cellulose (HEC), Avicel (PH101), laminarin, xylan from birch wood, locust bean gum (LBG), *p*NP (*p*-nitrophenol)-β-D-cellobioside, *p*NP-β-D-glucopyranoside, IPTG, porcine trypsin and bovine chymotrypsin were all purchased from Sigma-Aldrich (Oakville, ON, Canada). pCR4Blunt-TOPO vector kit and Pfx Ultra DNA polymerase were from Invitrogen (Burlington, ON, Canada). Restriction enzymes and T4 DNA polymerase and ligase were from Fermentas (Burlington, ON, Canada**)**. All other chemicals were of analytical grade and were obtained from either Sigma-Aldrich or Fisher Scientific (Nepean, ON, Canada). The regenerated amorphous cellulose (RAC) was prepared from the avicel (PH101) as previously described by Zhang *et al*.^61^.

### Animal feeding experiment and digesta collection

The crystalline cellulose pig feeding experiment for the induction of gut microbial cellulase genes had been conducted as described previously^23^. The induction diet including 10% crystalline cellulose of Solka-Floc^®^ as the sole fiber source was adapted from Rideout *et al*. with modifications^21^. The details of this diet formulation have not yet been reported, and are now supplemented here for the first time (Table S2).

### Construction of metagenomic expression plasmid library

Metagenomic DNA extraction, expression library construction and functional screening were performed as described previously^23^. The DNA insert size determination and the activity-based functional screening were further illustrated in the Supplemental Materials (Fig. S1). The inserts in positive clones  were sequenced by primer walking and assembled in the Molecular Super Center at the University of Guelph. The nucleotide sequence of insert in positive clone of p4818 was deposited in GenBank with an accession number of MH373350.

### Sequence and phylogenetic analyses

The possible open reading frames (ORFs) in the insert sequence were identified with the ORF finder at National Center for Biotechnology Information (NCBI; www.ncbi.nlm.nih.gov/projects/gorf/). Modularity and signal peptides of the enzymes were predicted by Simple Modular Architecture Research Tool (SMART; http://smart.embl-heidelberg.de). The characterized GH5 enzyme sequences (563, as of Mar. 3^rd^, 2019) were retrieved from the CAZy database (http://www.cazy.org/). The sequences were aligned using MUSCLE in Geneious 8.0.5 (Biomatters Ltd, New Zealand), and the sequences with fewer than 100 amino acids or that lack catalytic residues (i.e., equivalent to E314 and E227 in p4818Cel5_2A) were removed. The catalytic domains of the remaining 556 curated GH5 sequences were extracted and then realigned using Geneious 8.0.5 prior to constructing a neighborhood-joining tree using Geneious Tree Builder. The bootstrap analysis was chosen for resampling with a replicate number of 100.

### Protein expression and purification

The gene encoding the mature form of p4818Cel5_2A (with the signal peptide sequence of 1–23 amino acids removed) was amplified by PCR using the primers with the sequences of GGTCACATATGTTAACTCTGGGGATTATAATTG (forward) and CTCCAAGCTTCTAGTGCTGGTTCAGTATGTC (Reverse) (introduced NdeI and HindIII restriction sites are underlined). The amplified fragment was purified, digested and ligated into the vector pET28a, and fused in frame with an N-terminal His-tag for generating pET28a- p4818Cel5_2A using standard protocols^62^. The construct was verified by DNA sequencing in the Guelph Molecular Super Center.

Recombinant *E*. coli BL21 (λDE3) cells harboring the overexpression construct of pET28a-p4818Cel5_2A were propagated in 1 L of Luria-Bertani medium supplemented with 50 µg/mL kanamycin at 37 °C until optical density at 600 nm (OD_600nm_) reached 0.8. The culture was then induced by adding IPTG to a final concentration of 0.5 mM and followed by shaking at 200 rpm for 18 h at 15 °C. The cells were harvested by centrifugation at 5,000 × *g* for 10 min. The cell pellet was resuspended in a lysis buffer containing 300 mM NaCl, 10 mM imidazole and 50 mM sodium HEPES at pH 7.0 and disrupted by a French Press three times at an operating pressure of 12000 psi. The cell debris was removed by centrifugation at 17,500 × *g* for 10 min. Unless stated otherwise, the buffer used for affinity chromatography is 50 mM HEPES containing 300 mM NaCl at pH 7.0. The supernatant was filtered through a 0.45-µm filter and mixed with the Ni-NTA resin for 40 min with the gentle stirring under 4 °C. The mixture was poured into a column and the resin was washed with 300 ml of buffer containing 20 mM imidazole. The protein product p4818Cel5_2A was eluted with a buffer containing 250 mM imidazole. The fractions with enzyme activity were pooled and dialyzed overnight with the storage buffer of 50 mM HEPES containing 5% (v/v) glycerol, pH 7.0, and then concentrated with Pierce protein concentrator (10 K; MWCO; Fisher Scientific; Ottawa, ON, Canada) to a final concentration of 3.5 mg/ml. The purified enzyme was aliquoted and stored at −80 °C.

### Enzyme activity assay

All enzyme activity assays were performed in triplicate. The hydrolysis activity on various polysaccharides was examined by measuring reducing sugars released from substrates with the dinitro-salicylic acid (DNS) method^63^. Standard enzyme assay solutions contained 5.0 mM DTT and 1.0% (wt/vol) (10.0 mg/ml) of polysaccharide in 0.50 ml of 100 mM MES buffer (pH 6.0). The reaction was initiated by the addition of an amount of enzyme determined to release products in a linear relation to time when incubated at 50 °C for up to 10 min. The reaction was terminated by adding 1.5 ml of DNS solution and followed by incubation of 10 min under 100 °C to develop the color. The absorbance at 540 nm was measured using Pharmacia Ultrospec 2000 UV/VIS Spectrophotometer. One enzyme activity unit (U) was defined as the amount of enzyme that releases 1.0 μmole of D-(+)-glucose equivalent from the tested substrate per minute under activity assay conditions. D-(+)-glucose was used to generate a standard curve.

The p4818Cel5_2A activity on 4-nitrophenyl (*p*NP) glycosides was determined by measuring *p*NP release^61^. The typical reaction mixture contained 1.0 mM of the substrate in 0.5 ml of 100 mM MES buffer (pH 6.0). After the reaction was initiated by the addition of enzyme, the reaction mixture was incubated at 50 °C for 10 min. The reaction was terminated by adding 1.0 ml of 4.0% (wt/v) Na_2_CO_3_ to the mixture. The amount of *p*NP formed was measured by the A_405nm_ (extinction coefficient ε[405 nm] = 17,600 M^−1^ cm^−1^), and *p*NP was used to generate a standard curve. As indicated above, the enzyme dosage added to each assay mixture was optimized to measure initial reaction rates.

The kinetic assays were carried out under standard conditions except that the substrate concentrations were varied from at least 0.1 *K*_m_ to 5 *K*_m_. The collected data were fitted by the nonlinear regression analysis to the Michaelis-Menten equation using Sigmaplot 10.0.

The hydrolysis pattern of the enzyme was also investigated by monitoring specific viscosity decrease in a 3.0% (wt/vol) (30.0 mg/ ml) CMC solution using a controlled strain rheometer (MCR 301, Anton Paar GmbH, Ostfildern, Germany) equipped with a Peltier temperature controller and a parallel plate geometry (PP50, d = 50 mm, Anton Paar) was used. The purified enzyme of 19.3 μg was added to 30 mg/ml CMC solution in 100 mM MES buffer at pH 6.0 to trigger the reaction in a final volume of 16 ml, and the viscosity and reducing sugar concentration of the reaction solution were monitored at 2.0 and 5.0 min intervals, respectively, for 1.0 h.

### Optimum enzyme reaction conditions, enzyme stability, and effects of divalent ions

The effect of pH on enzyme activity was determined over a pH range from 4.0 to 10.0 with increments of 0.5 pH-unit using a constant ionic strength buffer containing 100 mM Tris, 50 mM acetic acid and 50 mM MES. The same buffers were used to examine the pH effect to the stability of enzyme activity, and the enzyme was incubated at different pH levels at 4 °C for 24 h prior to the activity assay. The temperature effect on enzyme activity and stability was assessed at different temperatures ranging from 15 to 70 °C in 50 mM HEPES buffer, pH 7.0. The incubation time under different temperatures was 60 min. CMC 1.0% (wt/vol) was used as the substrate for the standard enzyme assays described above.

### Resistance of the enzyme activity to trypsin and chymotrypsin digestion

The p4818Cel5_2A or BamCel5 (Megazyme, E-CELBA) in the final concentration of 50 µg/ml was incubated with either 5000 U/ml trypsin [the Sigma/Aldrich BAEE enzyme activity unit using Na-benzoyl-L-arginine ethyl ester (BAEE) as a typical assay substrate] or 200 U/ml chymotrypsin [the Sigma/Aldrich BTEE enzyme activity unit using N-benzoyl-L-tyrosine ethyl ester (BTEE) as a typical assay substrate], respectively, at 37 °C in 100 mM MES buffer (pH 6.0). The residual cellulase activity was monitored at different incubation periods by the standard activity assay with CMC as a substrate.

### Cellulase processivity and hydrolysis product analysis

Cellulase processivity was presented as the ratio of soluble reducing ends to insoluble reducing sugar ends produced in assay reaction when using the regenerated amorphous cellulose (RAC) as a substrate^45^. Briefly, 1% (wt/vol, 10.0 mg/ml) RAC in 0.4 mL 100 mM MES buffer pH 6.0 (8.0 mg FP/ml) was incubated with 0.43 nmol p4818cel5_2A (18.8 μg) under 35 °C for the incubation time of 30, 60 and 90 min, respectively. The reaction was terminated by adding 0.05 ml 2.0 M NaOH and heat treatment at 70 °C for 10 min to ensure inactivation of the enzyme, and then neutralized using 0.05 ml of 2.0 M HCl. After centrifugation at 6900 × *g*, 10 min, the supernatant was quickly transferred and its reducing sugars were measured by DNS method with D-glucose as a standard as aforementioned^63^. The insoluble pellet was washed with 6.0 M guanidine HCl in 0.6 mL twice to remove any bound protein first and further with 100 mM MES buffer (pH 6.0) twice. MES buffer 0.5 mL was added back to washed filter paper disc. The remaining reducing ends in this insoluble fraction were then measured with DNS reagent. The hydrolysis products in the supernatant were analyzed with thin-layer chromatography (TLC) on Silica Gel 60 aluminum plates (Merck, Darmstadt, Germany), which was developed in ethyl acetate/water/methanol (8:3:4, v/v/v). The plate was sprayed with a diphenylamine-aniline stain^64^, which was made by dissolving 2 g of diphenylamine and 2 ml of aniline in 100 ml of acetone followed by the addition of 10 ml of concentrated phosphoric acid. After staining, plates were developed in an oven at 100 °C for up to 1 h.

### Protein assay

Protein concentrations were determined by the Bradford assay using bovine serum albumin as a standard. SDS-PAGE was performed and stained with Coomassie Blue according to established procedures^65^.

### 3-D Structure homological modeling

The 3-dimensional model of residue 91 to 384 of cellulase p4818Cel5_2A was generated by the SWISS-MODEL online server^66^ using the crystal structure of a homologous cellulase (PDB ID:1E5J) as a template^53^. Structure model was analyzed and visualized using PyMOL 2.1 (www.pymol.org).

## Supplementary information

Supplemental Materials

## Data Availability

All data generated or analyzed during this study are included in this published article and its Supplementary Information Files.
