# Supplementary material for: A processive endoglucanase with multi-substrate specificity is characterized from porcine gut microbiota
Source: Sci Rep. 2019 Sep 20;9:13630. doi: 10.1038/s41598-019-50050-1 (PMC6754456; doi:10.1038/s41598-019-50050-1)
Supplement: Supplementary file 1 — Supplemental Materials [file 41598_2019_50050_MOESM1_ESM.pdf]

Supplemental Materials:

A processive endoglucanase with multi-substrate specificity is characterized from porcine gut microbiota

Weijun Wang[1], Tania Archbold[1], Joseph S Lam[2], Matthew S Kimber[2], and Ming Z Fan[1*]

[1]Departments of Animal Biosciences; and [2]Cellular and Molecular Biology, University of Guelph, Guelph, Ontario, Canada N1G 2W1

[*]To whom correspondence should be addressed. Email: mfan@uoguelph.ca

Table S1. Summary of reported *in vivo* degradability values and fractional degradation rates of the purified crystalline cellulose (Solka-Floc®) and guar gum in growing pigs as well as the *in situ* maximal degradability values and fractional degradation rates of the purified crystalline cellulose (Solka-Floc®) and commonly used feeds in cows

Animal species (fibre sources)	Gastrointestinal site	Degradability (%)	Retention time (h)	Fractional degradation rate (%/h)	References
Growing pigs					
Pigs (Solka-Floc®)	Distal ileal level	62.1 ± 8.4	1.6	38.8 ± 5.3	[1,2]
Pigs (Solka-Floc®)	Fecal level	82.5 ± 3.5	44.5	1.9 ± 0.08	[1,2]
Pigs (guar gum)	Distal ileal level	37.7 ± 8.4	1.6	23.6 ± 5.3	[1,2]
Pigs (guar gum)	Fecal level	87.7 ± 3.5	44.5	2.0 ± 0.08	[1,2]
Cows					
Cows (Solka-Floc®)	Rumen	75.5 ± 1.5	48.0	1.2 ± 0.1	[3,4]
Cows (dry hay)	Rumen	69.7 ± 2.6	34.6	1.4 ± 0.1	[3]
Cows (haylage)	Rumen	85.0 ± 4.1	27.6	1.3 ± 0.2	[3]

Cows (DDGS)	Rumen	109.8 ± 10.4	69.0	1.1 ± 0.1	3
Cows (SBM)	Rumen	121.1 ± 11.0	36.8	1.1 ± 0.2	3

The compiled *in vivo* degradability values (%) of the purified crystalline cellulose (Solka-Floc®) and guar gum in this table in the growing pigs are from a previously reported study (1). Fractional degradation rates (%/h) of the purified crystalline cellulose (Solka-Floc™) and guar gum in the growing pigs are calculated by division of the reported degradability values[1] over the reported average retention time of feeds[2]. The compiled *in situ* degradability values (%) for the purified crystalline cellulose (Solka-Floc®) in the cow was the maximal *in situ* degradability (%) estimated from the linear relationship between *in situ* degradability (%) and incubation time (x, h) in the dry cow, $y = 20.32 + (1.15 \pm 0.06)x$, by assuming the fibre feed particle retention time in the rumen at 48 h by referring to the literature reports (3, 4). The compiled *in situ* degradability values (%) of the other common feeds are the maximal *in situ* degradability values (%) estimated from the curve-linear relationships between *in situ* degradability (%) and incubation time (x, h) measured in the dry cow (3). The corresponding ruminal retention time (h) is the retention time required to reach the maximal *in situ* degradability (%) for the corresponding feeds and is calculated as = the time to reach the half maximal degradability (T_{50}) x 2 (3). The *in situ* fractional degradation rates (%/h) of the purified crystalline cellulose (Solka-Floc®) and the common feeds in the cows were obtained through the linear regression analysis and the curve-fitting[3]. DDGS, distillers dried grains with solubles; and SBM, soybean meal.

Table S2. The composition of the experimental diet for inducing the crystalline cellulose degradation microbiota in the growing pigs

Ingredient	Diet composition (g/kg diet)	Calculated nutrient content	
Poultry meal	450.0	DE, MJ/kg	157.4
Casein	40.0	Crude protein, g/kg diet	371.7
Animal fat	150.0	Ca, g/kg diet	10.90
DL-methionine	3.9	P, g/kg diet	9.9
Sucrose	43.1		
Cornstarch	200.0		
Cellulose (Solka-Floc^{TM})	100.0		
Vitamin-mineral premix	4.0		
Iodized salt	5.0		
Titanium dioxide	3.0		

The formulation of the diet was adapted from our previous studies[1]. Titanium dioxide is an alternative nutrient digestibility marker. The dietary nutrient contents were calculated according to the NRC (1998)[5].

Table S3. Effects of selected cationic ions and chemical reagents on the cellulase activity of purified P4818Cel5_2A

Cationic ions or reagents	Residual relative activity (%)
Control	100.0 ± 2.1
Ca^{2+} (1.0 mM)	102.2 ± 1.1
Co^{2+} (1.0 mM)	102.4 ± 3.8
Mg^{2+} (1.0 mM)	92.6 ± 2.5
Ni^{2+} (1.0 mM)	88.2 ± 2.4 [a]
Cu^{2+} (1.0 mM)	87.6 ± 0.5 [a]
Mn^{2+} (1.0 mM)	69.9 ± 1.9 [a]
Cd^{2+} (1.0 mM)	65.8 ± 0.8 [a]
Zn^{2+} (1.0 mM)	42.5 ± 0.9 [a]
DTT (5.0 mM)	127.4 ± 7.7 [a]
EDTA (5.0 mM)	93.7 ± 4.0
H_2O_2 (5.0 mM)	84.9 ± 5.1 [b]

Cellulase activities in the experiment were measured with the purified p4818Cel5_2A cellulase of 1.5 µg and 1% (w/v) carboxymethyl cellulose (CMC) as the substrate at the optimal pH of 6.0 and the optimal temperature at 50 °C for 10 min. Other details of the cellulase activity assay were described in the Material and Methods section.

Values are means ± SE (n = 3). The residual relative cellulase activities were expressed as % of the mean specific activity of the control group of 1816.0 ± 149.0 µmol D-glucose equivalent • μmol^{-1} protein • min^{-1}.

Different letters (a, b) indicate significant differences resulting from the effects of the selected cationic ions and chemical reagents on the cellulase activity of purified P4818Cel5_2A from the control, where a: $P < 0.05$ and b: $P < 0.01$.

Table S4. The comparison of ten cellulases used in the amino acid sequence alignments (see Fig. S5).

Enzyme name	Domain architecture	Processivity	Substrate profile	Reference
p4818Cel5_2A	Monomodular (GH5_2)	processive	CMC, avicel, solka-Floc®, RAC, β-(1,4)/(1,3)-glucan, xyloglucan, glucomannan and galactomannan	This study
BagCel5A	GH5_2-CBM5	ND[a]	CMC, RAC, Lichenan	[6]
EchCel5(EGZ)	GH5_2-CBM5	ND[a]	CMC, RAC,	[7]
PhaCel5G	GH5_2-(TSP-3)$_3$-CBM5	ND[a]	ND[a]	[8]
BsuCel5A	GH5_2-CBM3	processive	CMC, avicel, β-(1,4)/(1,3)-glucan	[9]
SdeCel5H	GH5_2-CBM6- CBM81	processive	CMC, avicel, β-(1,4)/(1,3)-glucan	[10]
BsuEG5C	GH5_2-CBM3a	processive	CMC, avicel, filter paper, RAC and β-(1,4)/(1,3)-glucan	[11]
VvoEG1	CBM1-GH5-5	processive	CMC, avicel, filter paper, RAC and β-(1,4)/(1,3)-glucan	[12]
HchCel5A	GH5_2-CBM6- CBM6	processive	CMC, avicel, filter paper, RAC and β-(1,4)/(1,3)-glucan	[13]
TmaCel5A	Monomodular (GH5_23)	processive	CMC, avicel, RAC and β-(1,4)/(1,3)-glucan.	[14]

a: ND represent not determined.

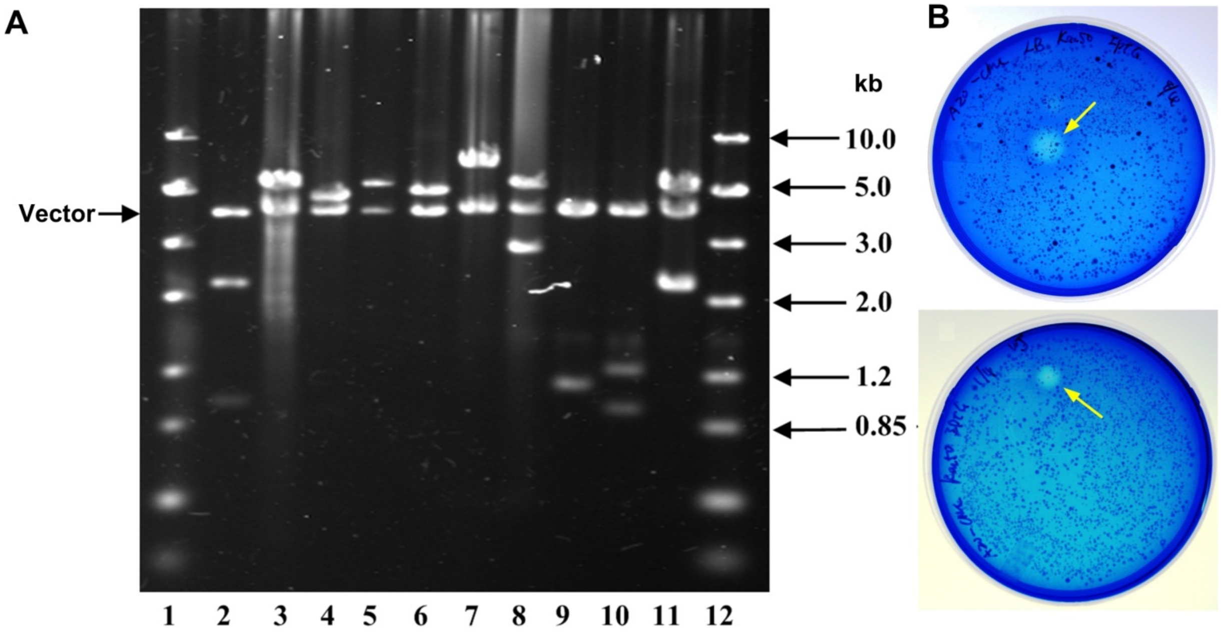

Fig. S1. Metagenomic plasmid expression library construction and functional screening. (*A*) Agarose gel electrophoresis (1.0%) of EcoR I digested plasmid DNA from ten randomly selected clones showing different insert size. The average insert size was approximately 4.6 kb. Lane 1, 12: DNA ladder; and lane 2-11: EcoR I-digested plasmid DNA. (*B***)** The activity screening against metagenomic expression library using AZO-CM-Cellulose. The yellow arrows indicated the colonies with carboxymethyl cellulolytic activities. The screening was performed at 37 °C for 48 h using LB agar plates containing 0.5 mM IPTG, 50 µg/ml kanamycin and 0.2 % chromogenic AZO-carboxymethyl cellulose (CMC) as a substrate.

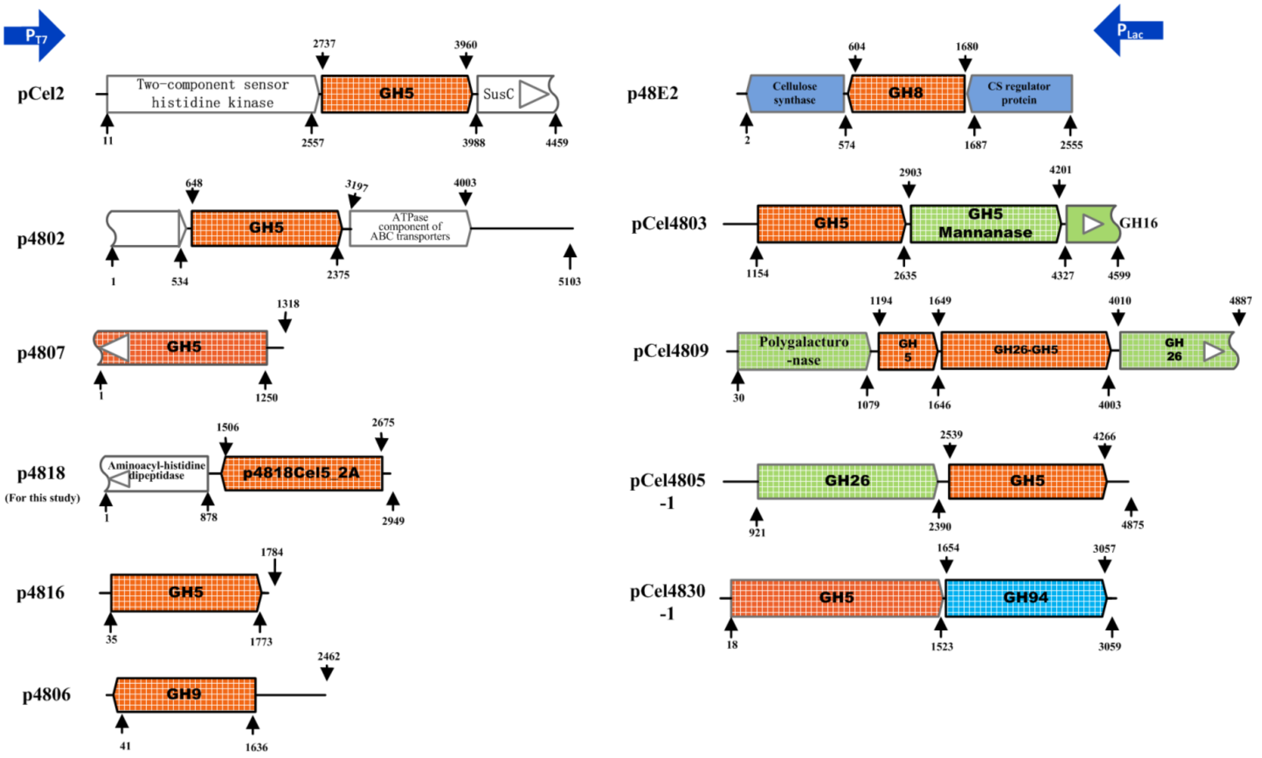

Fig. S2. The updated genomic organization of identified inserts from positive clones. Four function-related gene clusters were found in pCel4803, pCel4809, pCel4805-1, and pCel4830-1 with the glycoside hydrolase (GH) family annotation based on the Carbohydrate Active Enzymes database (http://www.cazy.org/). The predicted cellulase were boxed in orange.

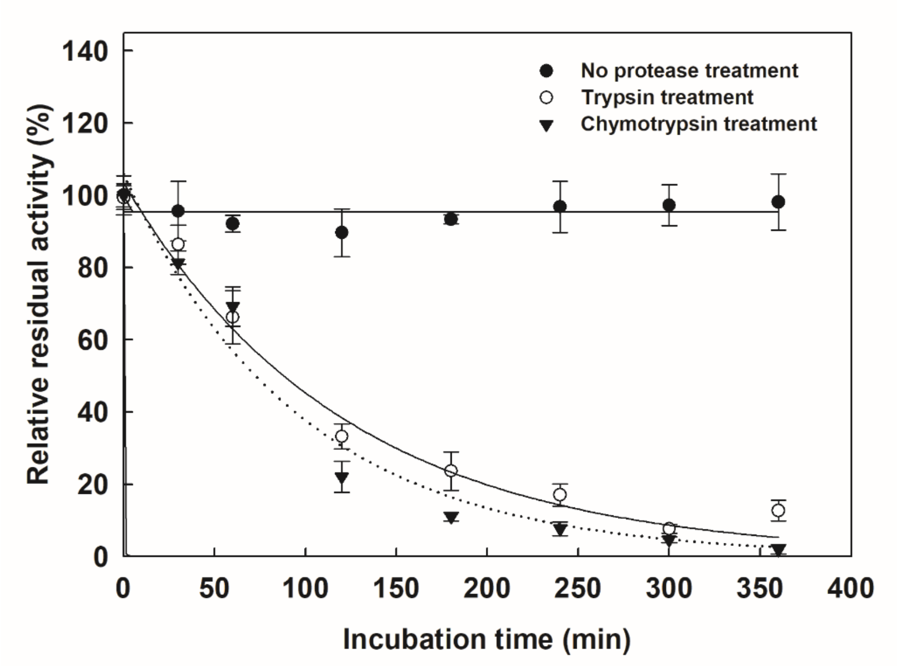

Fig. S3. Resistance of BamCel5 activity to porcine trypsin and bovine chymotrypsin treatment. BamCel5 was incubated with either 5000 U/ml trypsin (BAEE unit) or 200 U/ml chymotrypsin (BTEE unit) at 37 °C in 100 mM MES buffer (pH 6.0) in a final concentration of 50 μg/ml. The BamCel5 of 1.67 μg, and 1.0 % carboxymethyl cellulose (CMC) were used in the assays. Values were expressed as means ± SE, n = 3. The BamCel5 activity measured without any protease treatments was taken as 100%.

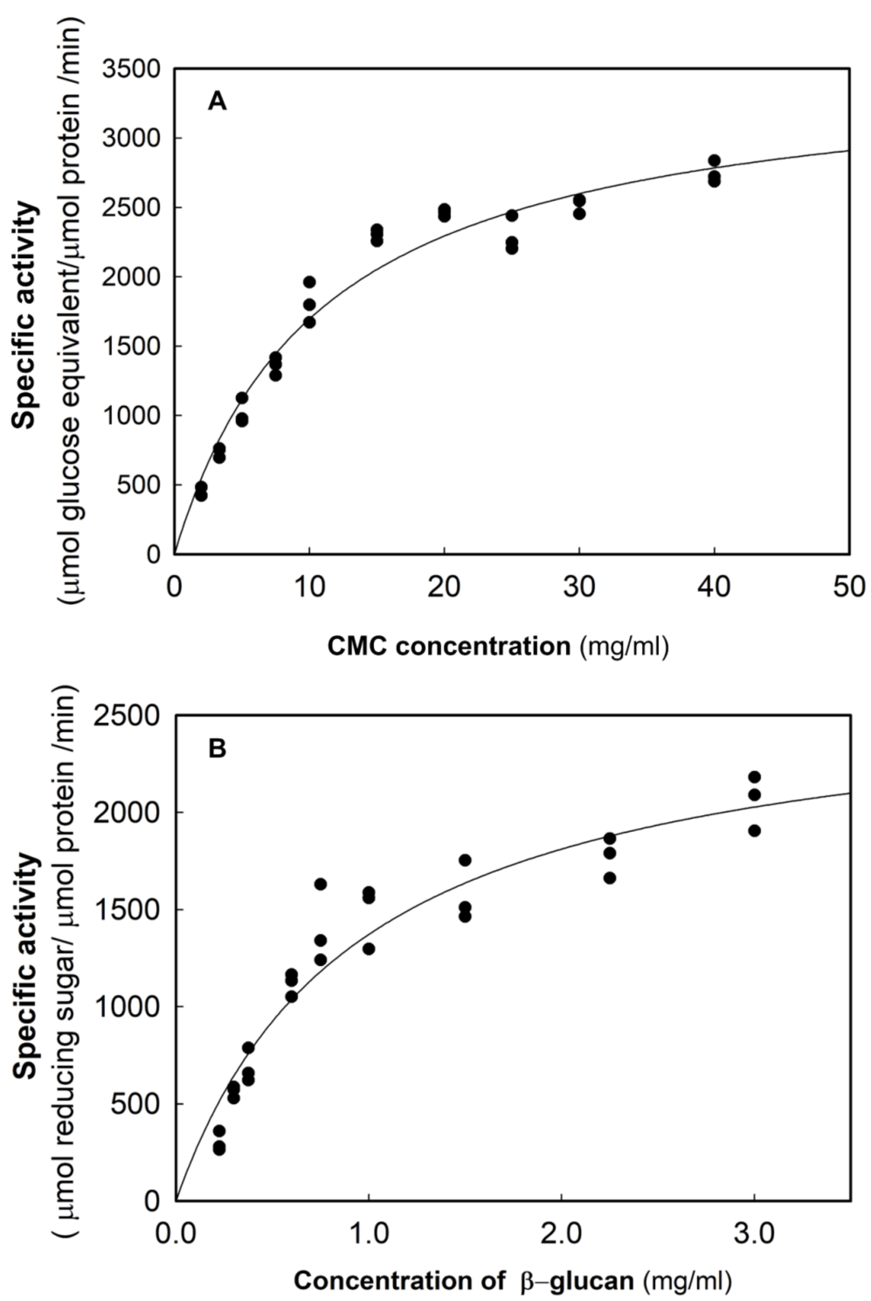

Fig. S4. Kinetics of the p4818Cel5_2A activity on carboxymethyl cellulose (CMC) and β-glucan from barley. (a) CMC was used as a substrate, K_m: 10.9 ± 1.1 mg/ml; and V_{max}: 3540.0 ± 130 µmol glucose equivalent • µmol^{-1} protein• min^{-1}; **(b)** β-Glucan was used as a substrate, K_m: 0.94 ± 0.15 mg/ml; and V_{max}: 2664.0 ± 177.0 µmol glucose equivalent • µmol^{-1} protein• min^{-1}.

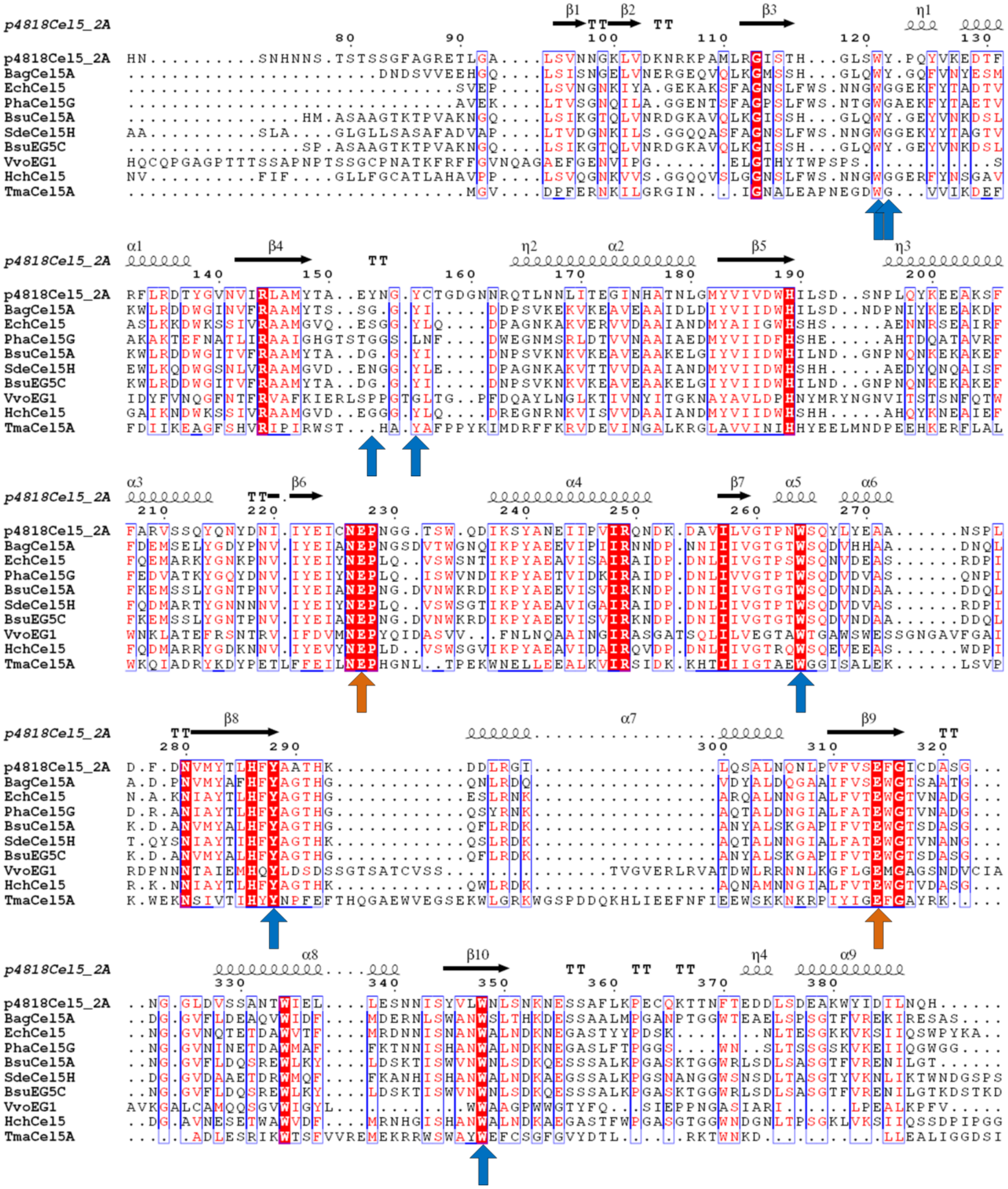

Fig. S5. The partial sequence alignments of the p4818Cel5_2A from this study with its relevant GH5 cellulases reported in the literature. Fully conserved residues are shaded in red.

The residues with the global similarity scores over 0.7 calculated using Reiser are boxed in blue. The η symbol refers to a 3_{10}-helix. The orange arrows indicate putative catalytic residues. The blue arrows indicate the aromatic amino acid residues that are predicted to be involved in cellulose binding. The sequence alignment was performed using ClustalW2 and the figure was prepared using ESPript and then modified manually. BagCel5A is an endoglucanase from from *Salipaludibacillus agaradhaerens* (previously called *Bacillus agaradhaerens*) (PDB#:1E5J)[6]; EchCel5 is a cellulase Cel5 from *Erwinia chrysanthemi* (PDB#:1EGZ)[7]; PhaCel5G is a cellulase Cel5G from *Pseudoalteromonas haloplanktis* (PDB#:1TVN)[8]; BsuCel5A is a processive endo-1,4-beta-glucanase from *Bacillus subtilis* 168 (PDB#: 3PZT)[9]; SdeCel5H is a processive endo-1,4-beta-glucanase from *Saccharophagus degradans*[10]; BsuEG5C is a processive GH5 endoglucanase (EG5C) from *Bacillus subtilis* BS-5[11]; VvoEG1 is a processive GH5 endoglucanase (EG1) from *Volvariella volvacea*[12]; HchCel5 is a processive GH5 endoglucanase (Cel5) from *Hahella chejuensis* KCTC 2396[13]; and TmaCel5A is a processive cellulase (Cel5A) from *Thermotoga maritima*[14].

References:

1. Rideout, T. C., Liu, Q., Wood, P. & Fan, M. Z. Nutrient utilisation and intestinal fermentation are differentially affected by the consumption of resistant starch varieties and conventional fibres in pigs. *Br. J. Nutr.* **99,** 984–992 (2008).
2. Navarro, D. M. D. L., Bruininx, E. M. A. M., Jong, L. De & Stein, H. H. The contribution of digestible and metabolizable energy from high-fiber dietary ingredients is not affected by inclusion rate in mixed diets fed to growing pigs. *J. Anim. Sci.* **96,** 1860–1869 (2018).
3. Fan, M. Z. *Concepts of Animal Nutrition, the 2nd Custom Edition*. (Pearson, 2015).
4. Yang, W. Z. & Beauchemin, K. A. Increasing the physically effective fiber content of dairy cow diets may lower efficiency of feed use. *J. Dairy Sci.* **89,** 2694–2704 (2006).
5. National Research Council. *Nutrient Requirements of Swine*. (National Academies Press, 1998).
6. Fort, S. *et al.* Mixed-linkage cellooligosaccharides: A new class of glycoside hydrolase inhibitors. *ChemBioChem* **2,** 319–325 (2001).
7. Chapon, V. *et al.* Type II protein secretion in gram-negative pathogenic bacteria: the study of the structure/secretion relationships of the cellulase Cel5 (formerly EGZ) from *Erwinia chrysanthemi*. *J. Mol. Biol.* **310,** 1055–1066 (2001).
8. Violot, S. *et al.* Structure of a full length psychrophilic cellulase from *Pseudoalteromonas haloplanktis* revealed by X-ray diffraction and small angle X-ray scattering. *J. Mol. Biol.* **348,** 1211–1224 (2005).
9. Santos, C. R. *et al.* Dissecting structure–function–stability relationships of a thermostable GH5-CBM3 cellulase from *Bacillus subtilis* 168. *Biochem. J.* **441,** 95–104 (2012).
10. Watson, B. J., Zhang, H., Longmire, A. G., Young, H. M. & Hutcheson, S. W. Processive endoglucanases mediate degradation of cellulose by *Saccharophagus degradans*. *J. Bacteriol.* **191,** 5697–5705 (2009).
11. Wu, B. *et al.* Processivity and enzymatic mechanism of a multifunctional family 5 endoglucanase from *Bacillus subtilis* BS-5 with potential applications in the saccharification of cellulosic substrates. *Biotechnol. Biofuels* **11,** 1–15 (2018).

12. Zheng, F. & Ding, S. Processivity and enzymatic mode of a glycoside hydrolase family 5 endoglucanase from *Volvariella volvacea*. *Appl. Environ. Microbiol.* **79,** 989–996 (2013).

13. Ghatge, S. S. *et al.* Characterization of modular bifunctional processive endoglucanase Cel5 from *Hahella chejuensis* KCTC 2396. *Appl. Microbiol. Biotechnol.* **98,** 4421–4435 (2014).

14. Basit, A. & Akhtar, M. W. Truncation of the processive Cel5A of *Thermotoga maritima* results in soluble expression and several fold increase in activity. *Biotechnol. Bioeng.* **115,** 1675–1684 (2018).
